# Wally Neuzil : a special woman and a therapist

**DOI:** 10.1080/17571472.2015.1113712

**Published:** 2015-12-24

**Authors:** Francesco Carelli

**Affiliations:** ^a^ University of Milan

Wally Neuzil was Egon Schiele’s companion for just four years. She was also the model who posed for many of his drawings and watercolors. Thinking about this woman, there is one thing that is particularly striking: it is her gaze – her strangely reticent, quietly searching gaze. It emanates from her bright, open eyes, barring the way to nothing and no one, perhaps almost seen as light emerging from them. Through her gaze she conveyed not only an inner calm, but also a mirror that neither distorted nor suppressed.

Wally had a quiet power – something we can still see today in the eyes of the portraits that Schiele created of her.

The exhibition that Leopold Museum in Vienna dedicated to Wally’s biography and her professions describes the fate of a woman at the *fin de siècle living* a life without taboos and with a profound humanity.

Wally Neuzil was not only Egon Schiele’s model from early 1911 but also his lover and faithful companion until the spring of 1915. While a model for Schiele, Wally also worked as a sales assistant, a cashier and mannequin at a clothing store.

## Wally’s key role in the life of Egon Schiele

Having started out as one of many models, Wally soon played a key role in Schiele’s life and works. Schiele created himself, his vision of an artist, through his works, while Wally revealed to him a world that was necessary for this development – an open sexuality that had slowly and gradually progressed. It was his route to a more stable, reliable self and an ability to have secure relationships. Through her quiet and strong presence, Wally also gave Scheile a clear and realistic mirror, encouraging the self-reflection that was indispensable and characteristic part of his work.

In the spring of 1911, Wally went with Schiele to Krumau. There, they lived together, in the conservative perspective of the time, ‘out of wedlock’. The fact that Schiele had young girls pose for him quickly became known within the small town, causing the local population to treat the two of them with increasing hostility.

That same summer, they were forced to leave Krumau. Schiele moved to Neulengbach, where Wally visited him in August 1911. But the couple faced difficulties there as well. A girl ran away from home and sought shelter with Schiele and Wally, spending the night at their house. This led to police searching Schiele’s studio, where they seized some erotic drawings. Schiele was remanded in custody and forced to spend 24 days in prison between April and May 1912. Wally stood by him during those difficult days, trusting in his integrity and providing active support throughout this crisis. Schiele was then convicted of distributing indecent drawings in St. Pölten and sentenced to three more days in prison, which were added on to the time he had already served. Heinrich Benesch picked Schiele up from prison the day he was released. Both his mother and Wally were waiting for him at the train station. In a letter to the collector Franz Hauer, Schiele wrote in 1914:

‘Among my closest acquaintances nobody did anything, except for Wally, whom I had only recently met and whose conduct was so noble that I was captivated’.

## Edith Harms: the separation

When Schiele met the sisters Edith and Adele Harms, his relationship with Wally was thrown into a deep crisis. The Harms sisters were living in the house opposite Schiele’s studio at Hietzinger Hauptstrasse. In 1915, Schiele eventually separated from Wally in a devastating scene and decided to marry Edith Harms, who came from a middle-class background. The fact that Schiele was drafted into the Army, as a soldier was certainly one of the reasons behind this speedy wedding. Wally Neuzil overcame her crisis and decided for her part to return confidently and actively to society.

She trained to be a nurse and worked in a war hospital in Vienna. In 1917, she volunteered to go to Dalmatia, where she died towards the end of that year from scarlet fever aged only 23. Schiele himself died in December 1918 of Spanish flu.

## Wally and Egon merged

At the centre of this exhibition is not just any woman, let alone one of these women belong to the artist’s studio and form a partnership with him, but rather a very specific woman. Wally Neuzil was indispensable for Schiele’s manner of working and became inextricably linked with his work. She was not a shadow being, but rather a light that Schiele needed for his work and self-reflection. Wally Neuzil was not only a model but also an understanding companion and a clear, realistic mirror. She fulfilled a role that one might ascribe today to a therapist, but clearly added an eroticizing libido. It calls to mind the relationship between Goethe and Charlotte von Stein. The fact that both artists left their important relationships when an evolution was complete is tragic but also logical. But for the same reason, it is understandable as to why these separations were so dramatic and difficult. Arguably, the most fascinating aspect of this relationship is the fact that Wally’s presence deepened Schiele’s awareness of himself and of his work. In the work ‘Prophets’ Wally appears as the missing link between Schiele as a person and as a prophet.

Within this evolution, Schiele also performed a symbolical gender swap: in the eminent oil painting ‘Caress (Cardinal and Nun)’, the ‘nun’ bears the traits of Schiele whilst from the work ‘Self-Portrait with Raised Bare Shoulder’ the ‘cardinal’ has Wally’s bare legs (as they appear in a watercolor created around the same time).

By giving Wally a mask, Schiele showed that he ascribed the role of a mediator, of a spiritual–physical catalyst to her. While the artist appearing clad like a nun was a daring sexual travesty far ahead of its time, the rendering also has spiritual meaning.

Wally Neuzil also benefitted from her relationship with Schiele, and perhaps even from their separation. She appreciated her companion as an artist and was, in turn, appreciated and taken seriously by him; something which strengthened her sense of self.

When he left her, she was not destroyed, but bravely carried on with her life.

The book accompanying the exhibition is thus not a catalogue with its conventional separation of art historical texts and illustrations, but rather a book in its own right, valid far beyond the duration of the exhibition. It is the result of work carried out by the creative team of the Leopold Museum [the Egon Schiele Documentation Centre] and close cooperation with Brandstätter publishers.

Many thanks to Klaus Pokorny, of the Leopold Museum in Vienna, for having provided me with the book of the exhibition.

